# Stress and Its Correlates in Migraine-Headache Patients with a Family History of Migraine

**DOI:** 10.3390/bs12030065

**Published:** 2022-03-01

**Authors:** Khalid Al-Quliti

**Affiliations:** Neurology Division, Department of Medicine, College of Medicine, Taibah University, Medina 42353, Saudi Arabia; kquliti@taibahu.edu.sa

**Keywords:** migraine, headache, sports, sleep, sleep duration, sleep deprivation

## Abstract

**Purpose:** Stress and migraine are often comorbid. However, no studies have examined stress severity in a sample of migraine patients. That is why this study investigated the determinants of stress level in a sample of migraine patients with a family history of migraine (MWFH) in Saudi Arabia. **Material and Methods:** A quantitative observational study with a cross-sectional data collection and convenient sampling of (MWFH) was performed in Madinah, Saudi Arabia. Participants completed the Perceived Stress Scale-4 (PSS-4) and a list of items to register clinical history and demographics information. **Results:** Out of eight independent variables that were explored, only two variables —both sleep related, i.e., insufficient sleep (β = 0.22, *p* = 0.04) and non-refreshing sleep (β = 0.22, *p* = 0.04), *F*(8, 127) = 5.13, *p* < 0.001, *R*^2^ = 0.244—were associated with stress severity. The majority of (MWFH) were female (73.7%), recorded a lack of habitual physical activity (56.2%), received treatment for co-morbidities (56.9%), reported sleep insufficiency (54%), non-refreshing sleep (52.6%) and traumatic incidents (50.4%). **Discussion:** Stress severity increased with sleep complaints, indicating a comorbidity of stress–sleep problems among migraine patients with a family history of migraine.

## 1. Introduction 

Psychological or perceived stress is a situation in which individuals perceive their environment requires more resources than the person is capable of adjusting to [1]. Such a demand may risk health, well-being, and quality of life [1]. Stress is very common among patients who suffer from migraines, with about 70% reporting it initiates acute episodes [2]. In comparing frequency, stress scores as No. 1 among the known triggers of migraine episodes [3]. A more detailed examination of this stress–migraine relationship shows that these two share more intricate dynamics. Stress–migraine temporal alignment reveals three types of migraine patient clusters, each with a slightly distinct pattern [4]. If considered at the aggregated group level, stress level increases during the pain phase of migraine [4]. Psychological stress may also play a role as a predisposing factor in the etiology of new onset migraine [5]. Though many studies identify a positive association between stress and migraine, i.e., increasing stress usually increases the frequency and intensity of migraine; evidence of this relationship is not unanimous, with some studies indicating daily-life-related stress may not increase migraine [6]. Knowing this shows there is a clear need to further explore this relationship. Therefore, in this study, association between stress severity level and migraine was assessed.

The relationship between stress and migraine has a multidimensional aspect that may appear in adverse physical and psychological situations in affected individuals [7]. Co-morbid stress in cases of frequent migraines may promote development and exacerbation of additional health conditions and diseases. This stress may be cyclic in terms of cause-effect relationship of migraine and additional health conditions and diseases [7]. Furthermore, taking into consideration accumulated evidence from previous studies, few studies explore correlates or determinants of stress severity level in migraine patients. In males, lower age of migraine onset, more days taking migraine medication, and more days with migraine episodes were associated with a family history of migraines [8]. Similarly, in pediatric migraine patients, younger age of onset and longer duration of migraine episodes were associated with a family history of migraines [9]. These pieces of evidence show that some migraine characteristics are more common in patients having a family history of migraine [8]. Migraine patients with a family history of migraine seem to represent a separate cluster or subgroup within migraine patients, which is linked to genetic factors. Familial hemiplegic migraine, which is inherited in an autosomal dominant fashion, while out of all the linkage studies done on migraine the most consistent locus resides on chromosome 4 [10]. Data on migraines without aura showed robust evidence from epidemiological studies in twins, families, and unrelated cases of migraine indicating genetics plays a significant part in migraine expression. The results indicate that first degree relatives of migraineurs are RR = 1.88 times more likely to suffer migraines than first degree relatives of non-migraineurs [10]. Studies on monogenic migraines, a rare disorder, show a large impact on the patients and families involved. The majority of migraines are polygenic, a complex disorder with multiple variants in genes contributing to the underlying risk, with each variant having a relatively small effect [11].

Studies on migraine patients showed that the most common sleep disturbances are insomnia, daytime sleepiness, sleep apnea, and parasomnia which also has an impact on quality of life and is associated with increased disability [5,6].

Studies on personality traits in migraine showed that migraineurs display increased neuroticism and anxiety and are anti-aggressive. Also, some of these personality traits could be an effect of serious and insufficiently processed life events, as it is shown that migraine patients may have a history of maltreatment, especially during childhood. Studies reported increased neuroticism in both sexes in a longitudinal study on migraine subjects between 19 and 29 years of age. Farther more, an increase amount of neuroticism was associated with a higher incidence of migraine, which was more marked for female patients [4,5,6,7].

Therefore, research focused on understanding migraine epidemiology, correlates, and pathophysiology in this group may help in targeted management of their migraine-related problems. This study investigated the applicability of a model to predict changes in the stress severity level among clinically diagnosed (MWFH).

## 2. Material and Methods

### 2.1. Participants and Procedure

This was a quantitative observational study to investigate psychological stress level and determine its correlates in (MWFH). A cross-sectional approach to data collection was implemented on a convenient sample of (MWFH) who presented at the outpatient division of Ministry of Health hospitals in Madinah, Saudi Arabia. The study was carried out during a four month period from October 2019 to January 2020. Inclusion criteria were adults with migraine headache complaints for at least six months, based on the diagnosis criteria for migraine based on the International Classification of Headache Disorders, and also a family history of migraine. Exclusion criteria were having headache–migraine but not meeting any of these: (i) headache–migraine complaints for at least six months, (ii) no account/record of family members having migraine–headache, and (iii) under 18 years of age. Headache–migraine features in patients were based on the reported history, clinical evaluation, and medical records. Family history of migraine–headache was based on the subjective account of patients.

The researcher presented the questionnaire-based survey forms and clinical evaluation during the patients’ regular visits to the outpatient department. Patients were screened and those fulfilling inclusion criteria were asked to participate. All those deemed suitable to participate and who provided written consent to participate were further given a brief introduction about the aim and objective of the study in simple language.

Altogether, 137 migraine–headache patients with a family history of migraine (age: 27.3 ± 7.0 years) participated. Participants were required to respond to the study questionnaire at interviewer-administered sessions. The questionnaire package contained a shortened version of the Perceived Stress Scale known as PSS-4, and collected a clinical history and demographic information.

### 2.2. Perceived Stress Scale (PSS-4)

PSS is a widely used subjective tool to assess severity of psychological stress in clinical and research settings [12,13,14]. PSS has been validated in a number of settings and different types of populations including Afro-Asians [12,13,14]. A recent study also found it to have adequate psychometric properties such as internal consistency, convergent validity, divergent validity, and factorial validity in a sample of a Saudi population [14]. PSS-4 is a brief version of the scale with only four questions; all the questions are scored on a five-point Likert scale. The scores for two questions need to be reverse coded, and finally, a total score is calculated by adding the scores for all individual items. Higher scores indicate a more severe level of stress-related symptoms [12,13,14].

### 2.3. Clinical History and Socio-Demographics

A set of questions with both open- and closed-ended items was used to collect information related to social, demographic, and clinical history. The response to the question regarding clinical diagnosis of headache was based on participants’ medical records. In turn, this information was based on expert clinical diagnosis by neurologists at Madinah hospitals using the International Classification of Headache Disorders. Participants were required to record their age, gender, sports activity, treatment for other (apart from migraine-headache) medical conditions, recent traumatic accidents/incidents among family/friends, and sleep-related complaints, etc.

### 2.4. Statistical Analysis

Analysis was performed by the SPSS version 26.0. Percentage, mean, and standard deviation were estimated for presenting descriptive statistics. The multiple linear regression model was used to assess association between dependent variables, PSS-4 total score indicator of stress severity level, and independent variables. Demographic variables and clinical parameters were taken as independent variables.

## 3. Results

### 3.1. Participants’ Characteristics

The average values of age and PSS-4 total scores were 27.3 ± 7.0 years and 8.1 ± 2.5, respectively in the study sample (Table 1). Most participating (MWFH) had migraine without aura (66.4%). Nearly three-fourths of migraine patients with a family history of migraines (73.7%) were female. Most participants did not engage in regular exercise or sports activities (56.2%). The majority of participants were currently under treatment for comorbid medical conditions (56.9%) and most migraine patients with a family history of migraines complained of insufficient sleep (54%). A little less than half of the participants (MWFH) complained of not feeling refreshed after sleep (47.4%). A little more than half of the participating migraine patients reported a recent traumatic incident in the family, e.g., death of a relative or close friend (50.4%).

### 3.2. Multivariate Analysis: Multiple Linear Regression

**Figure 1:**
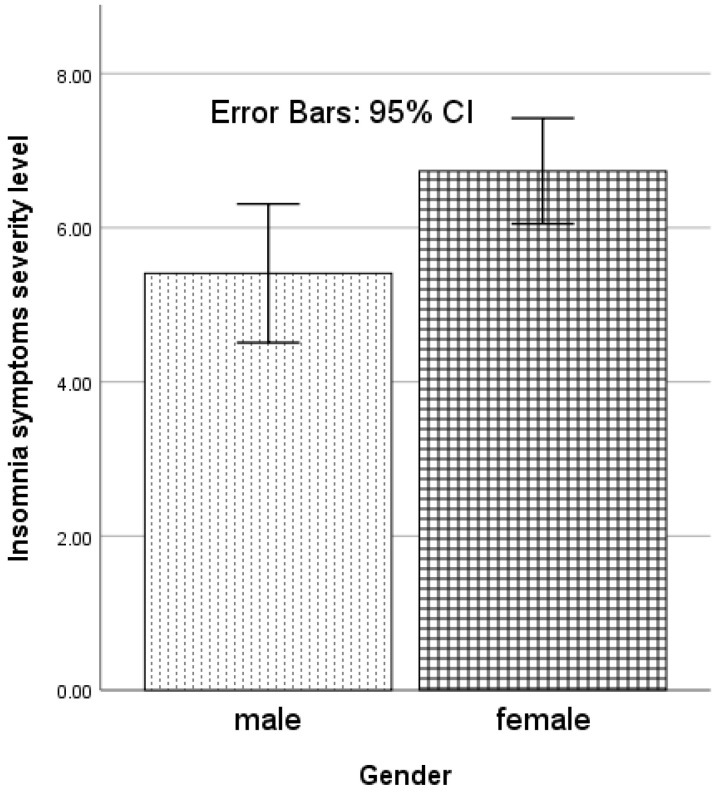
Gender-related variation in insomnia symptoms severity level (Athens Insomnia Scale score) in migraine patients with/without aura.

**Figure 2:**
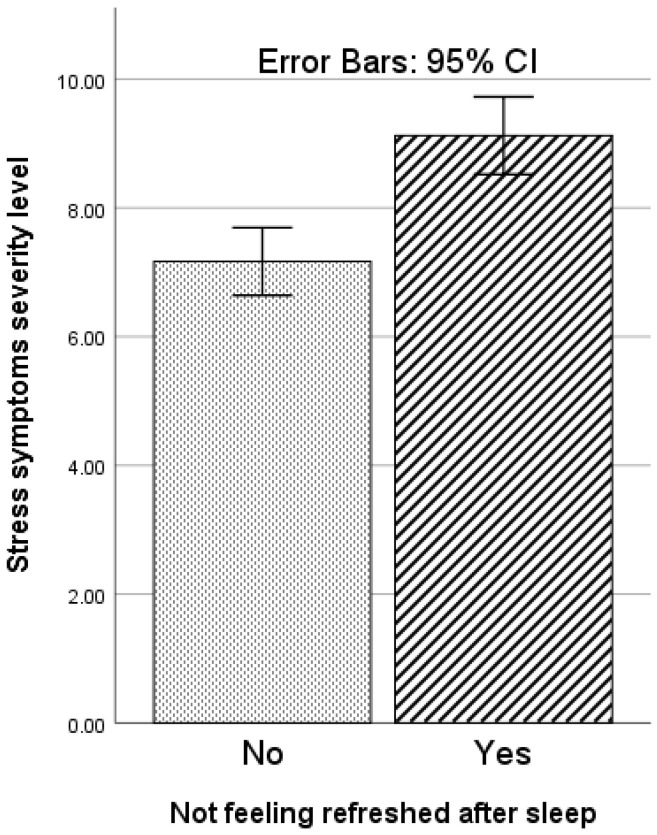
Variation with complaint of not feeling refreshed after sleep in stress symptoms severity level (assessed by PSS-4: Perceived Stress Scale-4) in migraine patients with a family history of migraines.

A multiple linear regression was run to predict changes in the stress level in migraine patients with a family history of migraines from clinical diagnosis (i.e., migraine with aura, migraine without aura, and chronic-tension type headache), gender, age, sports activity (Yes/No), currently under treatment for medical conditions (Yes/No), complaint of insufficient sleep (Yes/No), not feeling refreshed after sleep (Yes/No), and recent traumatic incident in the family, e.g., death of a relative or close friend (Yes/No). The study model significantly predicted changes in stress levels in the study sample, *F*(8, 127) = 5.13, *p* < 0.001, *R*^2^ = 0.244 (Table 2). Complaints of insufficient sleep (*p* = 0.042) and complaints of not feeling refreshed after sleep (*p* = 0.037) significantly predicted stress levels (Table 2). Those complaining of insufficient sleep had a higher level of stress (Figure 1). Similarly, those who complained of not feeling refreshed after sleep had a higher stress level (Figure 2).

## 4. Discussion

To the best of this researcher’s knowledge, this is the first study to investigate correlates of stress severity level in a sample of clinically diagnosed (MWFH). The findings of this study reveal increasing stress severity was predicted by sleep disturbances such as complaints of insufficient sleep and of not feeling refreshed after sleep. The majority of participants (MWFH) had migraine without aura, were females, participated in no regular sport or exercise activities, were undergoing treatment for co-morbid medical conditions, complained of insufficient sleep, and had a recent traumatic incident in the family.

A robust approach of multivariate analysis involving multiple linear regression found the model with eight predictor variables was statistically significant and explained about 24% of the changes in the variance level of stress severity. This finding reaffirms and adds to the accumulated evidence about the positive association between stress and migraine [6]. Rafique et al. understandably reasoned on the basis of the absence of an unequivocal line of evidence from epidemiological studies, that the stress–migraine relationship seems to be non-resolved [6].

However, if a collective appraisal is made using evidence from epidemiological studies, the outcome of this study, animal model investigations, and mechanistic studies, then most seem to support the stress-migraine relationship. Malone et al. reasoned the stress–migraine relationship may have implications for additional health conditions and diseases [7]. Indeed, findings from this study that showed two sleep problems: insufficient sleep and unrefreshing sleep, were associated with increasing severity of stress symptoms, and do seem to favour the thematic synthesis that stress in migraine involves sleep complaints [7]. Similarly, Houle et al. found that concurrent stress and poor sleep are usual risk factors of headache [15]. Sullivan and Martin found that short sleep duration is associated with both migraine and nonmigraine-related headache frequency [16]. Furthermore, Sullivan and Martin also found that sleep quality impacts the relationship between sensitivity to sleep insufficiency or lack of sleep and headache frequency [16]. There are no previous reports about non-refreshing sleep in migraine and its relationship to stress. However, some reports do support about other aspects of sleep being associated with stress in migraine [7,15,16].

In this study, the sample of migraine patients was comprised of three distinct diagnoses groups, of which migraine without aura, formed the biggest group. This is similar to previous reports about the overwhelming presence of migraine without aura group as the most common type of migraine Schramm et al., using the prevalence and predictor analysis of migraine that was used for cardiovascular events in the Heinz Nixdorf Recall study data, found that of all active migraine cases, migraine without aura formed one of the biggest groups [17].

In this study, the majority of participating (MWFH) were female. The fact more women get migraines is perhaps one of the most common findings from previous studies [17,18]. Pavlov et al. summarized that gender influence is prominent in the occurrence of migraines based on empirical evidence from four different groups of studies, namely epidemiology, clinical trials, animal models, and neuroimaging investigation [18].

In this study, the majority of (MWFH) had no regular exercise or sports activity. This is consistent with previous reports [19]. In a recent study, Rogers et al. found those with migraine report less physical activity [19]. In this study, the majority of participants (MWFH) were undergoing treatment for comorbid medical conditions. This finding is also similar to previous reports [20]. In a large national representative sample study in Scotland by McLean and Mercer (*n* = 1,468,404 adults), comorbidity was a predominant feature of migraine patients, with as many as 25 out of 31 health conditions being significantly higher among migraine sufferers [20,21].

Studies on gender differences were found in personality traits and in the number of stressful life events, in particular those rated as strongly negative. Furthermore, anxiety, depression, and tension-type headache were twice as frequent in women. It was advice that stress susceptibility, life events, and parallel disorders, especially of psychiatric nature, ought to be considered when investigating and treating individuals with migraine [22,23,24].

Studies on psychological/behavioural techniques for migraine and tension-type headache showed them to be effective for reducing headache frequency and intensity and improving sleep parameters such as sleep quality and sleep time [24,25].

In this study, the majority of participants (MWFH) had a recent traumatic incident in the family. Chowdhary and Enam discovered that migraine forms the biggest group of headache complaints among patients who have experienced traumatic incidents [21].

One noteworthy limitation of this study was the relatively small sample size of the study group, 137. This was done for feasibility, in light of the patient flow rate and confirmed diagnosis based of the International Classification of Headache Disorders in the participating facility. Though, the model explained about 1/4th of the variance in the level of the stress severity. This is quite substantial, however, there exists scope to further build on this finding. Future studies with a more extensive list of predictors may yield better models to account for even more variance levels in the changes of stress severity among migraine patients with respect to independent variables. This study relied on the word of migraine patients as to their family history of migraines. This approach may lead to an underestimation of migraine prevalence and incidence [22].

In brief, the outcome of this study showed that stress severity increases with sleep insufficiency and non-refreshing sleep in (MWFH). Additionally, the findings showed that among (MWFH), headaches occur mainly in females, those who lack habitual physical activity, have comorbid health conditions, suffer from sleep insufficiency, and have experienced traumatic incidents among family or friends.

## Figures and Tables

**Table 1:** Characteristics of participating headache/migraine patients with family history of migraine.

Characteristics	Mean ± SD/Frequency (Percentage)
Headache clinical diagnosis	
Migraine with aura	33 (24.1)
Migraine without aura	91 (66.4)
Chronic-tension type headache	13 (9.5)
Gender	
Male	36 (26.3)
Female	101 (73.7)
Age (year)	27.3 ± 7.0
Sports activity	
Yes	60 (43.8)
No	77 (56.2)
Currently under treatment for medical conditions	
Yes	78 (56.9)
No	58 (42.3)
Did not report	1 (0.7)
Complaint of insufficient sleep	
No	63 (46)
Yes	74 (54)
Not feeling refreshed after sleep	
No	72 (52.6)
Yes	65 (47.4)
Traumatic incident in the family, e.g., death of a relative or close friend	
No	68 (49.6)
Yes	69 (50.4)
PSS-4 score	8.1 ± 2.5

SD: standard deviation; PSS-4: perceived stress scale-4.

**Table 2:** Multiple regression predictors of the insomnia symptoms severity level in migraine patients with/without aura.

Independent Variable	Beta Coefficient	Standard Error	T Values	*p*-Values	Model Unadjusted *R*^2^; Adjusted *R*^2^; *p*-Value
BMI	0.19	0.11	1.97	0.05	0.17, 0.13, <0.01
Age	0.11	0.05	1.28	0.20	
Gender	0.25	0.70	2.81	0.01	
Migraine with-without aura	0.00	0.62	0.04	0.97	
Sports activity	−0.06	0.58	−0.77	0.44	
History of head-neck injury	0.05	0.58	0.62	0.54	
Currently under treatment for other medical conditions	0.16	0.61	1.91	0.06	
Frequency of migraine medicine intake	0.08	0.48	0.99	0.32	
Intercept	−3.43	2.91	−1.18	0.24	

Unstandardized beta coefficient for intercept, for all other independent variables standardized beta coefficient are shown. Insomnia severity was assessed by AIS: Athens Insomnia Scale.

## Data Availability

Not applicable.
